# Anatomical and Metabolome Features of *Haloxylon aphyllum* and *Haloxylon persicum* Elucidate the Resilience against Gall-Forming Insects

**DOI:** 10.3390/ijms25094738

**Published:** 2024-04-26

**Authors:** Nina V. Terletskaya, Aigerim Mamirova, Kazhybek Ashimuly, Yekaterina P. Vibe, Yana A. Krekova

**Affiliations:** 1 Faculty of Biology and Biotechnology, Al-Farabi Kazakh National University, Al-Farabi 71, Almaty 050040, Kazakhstan; kajeke@mail.ru; 2 Institute of Genetic and Physiology, Al-Farabi 93, Almaty 050040, Kazakhstan; 3 A.N. Bukeikhan Kazakh Research Institute of Forestry and Agroforestry, Kirov 58, Shchuchinsk 021704, Kazakhstan; wiebe_k@mail.ru (Y.P.V.); yana24.ru@mail.ru (Y.A.K.)

**Keywords:** *Haloxylon* spp., resistance, metabolome, anatomy, secondary metabolites

## Abstract

Globally, gall-forming insects significantly contribute to the degradation of desert ecosystems. Recent studies have demonstrated that *Haloxylon persicum* suffers less damage from gall-formers compared to *Haloxylon aphyllum*. However, the mechanisms driving the long-term metabolic responses of these species to gall-forming biotic stress in their natural environment remain unclear. The current study comparatively analyzes the anatomical features and metabolomic changes in *H. aphyllum* and *H. persicum* damaged by gall-forming insects. This research aimed to uncover potential metabolic tolerance mechanisms through GC-MS analysis. The study findings indicate that gall-forming insects cause a reduction in nearly all the anatomical structures of *Haloxylon* shoots, with the effects being less severe in *H. persicum* than in *H. aphyllum*. Thus, the metabolic pathways responsible for the biosynthesis of biologically active substances that enhance resistance to gall inducers were different, specifically in *H. aphyllum*—the biosynthesis of fatty acids (+their derivatives) and γ-tocopherol (vitamin E) and *H. persicum*—the biosynthesis of fatty acids (+their derivatives), dialkyl ethers, carbohydrates (+their derivatives), aromatic acid derivatives, phytosterols, γ-tocopherol (vitamin E), phenols, and terpenoids. The results suggest that the modulation of metabolic pathways under biotic stress plays a crucial role in the enhanced survival and growth of *H. persicum*.

## 1. Introduction

*Haloxylon* is a genus of woody plants belonging to the subfamily Chenopodioideae within the Amaranthaceae family. This genus comprises five species, three of which are native to Kazakhstan: *Haloxylon persicum* Bunge ex Boiss. & Buhse, *Haloxylon aphyllum* (Minkw.) Iljin, and *Haloxylon ammodendron* (C.A. Mey.) Bunge ex Fenzl. The habitats of the first two species largely overlap, covering the deserts of Central Asia and Kazakhstan. Meanwhile, *H. ammodendron* extends further east from Lake Zaisan into Mongolia and northwestern China [1,2]. In Kazakhstan, *Haloxylon* spp. are predominant over an area of approximately 6.73 million ha, accounting for 50.5% of the country’s forested lands. Most of this coverage is found in the Kyzylorda region, located in the southwest [3]. Within this region, the main forest-forming species are *H. aphyllum* and *H. persicum*.

*Haloxylon* spp. serve as crucial environment-forming edificatory species and are among the most economically valuable halophytes, performing multiple roles, including soil stabilization, provision of pasture forage, and a fuel source for local communities. By reducing wind speed, improving microclimatic conditions, and facilitating the colonization and growth of other desert plants, saxauls play a vital role in preserving the structure and functionality of the ecosystems they inhabit [4,5,6,7,8,9,10].

The saxaul forests of Kazakhstan are distinguished by their unique plant community composition, marked by a rich presence of ephemerals, ephemerids, wormwoods, and polycarpic species [11,12]. However, it is crucial to acknowledge the ongoing dynamics of natural changes in the floristic composition of these phytocenoses, alongside a decrease in the area of the most productive forests. These changes are attributed to a variety of abiotic and biotic factors. Consequently, identifying and examining the potential mechanisms for the resilience of saxaul ecosystems becomes increasingly important.

Pests play a substantial role in the degradation of desert ecosystems. The harmful insect fauna affecting saxauls includes a wide array of both polyphagous and specialized species, which inflict damage on the roots and vegetative and generative organs of these plants [13,14,15,16,17]. A variety of galls can be observed on the assimilated shoots, stems, and branches of *Haloxylon* spp. Worldwide, more than 17 species of gall inducers that damage saxaul forests have been identified [18,19,20,21,22].

In southern Kazakhstan, researchers have identified 213 forms of galls, induced by a range of organisms, including *Homoptera proboscis*, *Coleoptera*, *Hymenoptera*, *Diptera*, *Lepidoptera*, and mites. Among these, 71 forms were found on buds, 64 on shoots, 44 on leaves, 15 on flowers, 6 on seeds, and 1 on roots [23]. Notably, species such as *C. robusta*, *C. azurea*, *C. nana*, and *C. notata* from the genus *Caillardia* Bergevin were recorded for the first time in Kazakhstan and considered among the most detrimental, specifically affecting *H. aphyllum* and *H. persicum* [24,25,26].

Over the past decade, forestry institutions in the Kyzylorda region have reported widespread occurrences of gall-forming insect infestations. According to the 2023 statistical report from the Republican State Enterprise “Republican Forest Selection and Seed Center”, these outbreaks have affected an area of 97,817 ha. In regions where plants are damaged to varying degrees, there is a noticeable weakening of the plants, with significant reductions in growth, fruiting, and seed production observed. Currently, to manage the population of gall formers, various organizational, technical, agronomic, chemical, and biological strategies have been suggested [27]. However, in Kazakhstan, comprehensive scientific efforts to identify resistance markers and select saxaul plants resistant to these insects have yet to be undertaken.

Previous research has demonstrated that *H. persicum* sustains significantly less damage from gall formers compared to *H. aphyllum*. Notably, in the Kyzylkum desert, 20 species of gall midges have been identified on black saxaul (*Asiodiplosis*—9 species and *Stefaniola*—12 species), while white saxaul hosts 11 species (4 *Asiodiplosis* and 7 *Stefaniola* species) [2].

The literature suggests that gall formation is initiated by chemicals secreted by the gall inducer. However, the precise action mechanism of these chemicals and the overall process by which insects might control and manipulate plant development and physiology remain unclear. Moreover, gas chromatography-mass spectrometry (GS-MS) analysis has shown that some bioactive compounds in plants (such as lipids, in particular, unsaturated fatty acids, phenols, terpenoids, tocopherols, some alkanes, and ketones) may act as mediators in response to biotic and abiotic stresses [28,29,30].

Interestingly, plant galls have been identified as valuable sources for studying biologically active substances, which are significant due to their physiological and adaptive characteristics [31]. Metabolomic studies can shed light on physiological, biochemical, phenotypic, and morphological processes, including plant and microbial community responses to environmental changes and pathogen interactions [32,33]. Notably, plant metabolomics employing GS-MS has proven highly effective in analyzing the metabolism of forest species under stress conditions [34,35,36].

Nevertheless, the detailed mechanisms behind the long-term metabolic responses of *H. aphyllum* and *H. persicum* to gall-forming insects in their natural environment are still to be fully understood. Thus, conducting a comprehensive comparative analysis of the anatomical, morphological, and metabolomic alterations in *H. aphyllum* and *H. persicum* due to gall damage is proposed as a promising research direction. This could help identify potential metabolic mechanisms of tolerance in these species. Indeed, the study findings will contribute to significant enhancement of the understanding of insect–plant interactions and provide a scientific foundation for addressing the impact of gall formers on these valuable desert plants.

## 2. Results

### 2.1. Anatomical Structure of Intact and Damaged H. aphyllum and H. persicum

**Figure 1:**
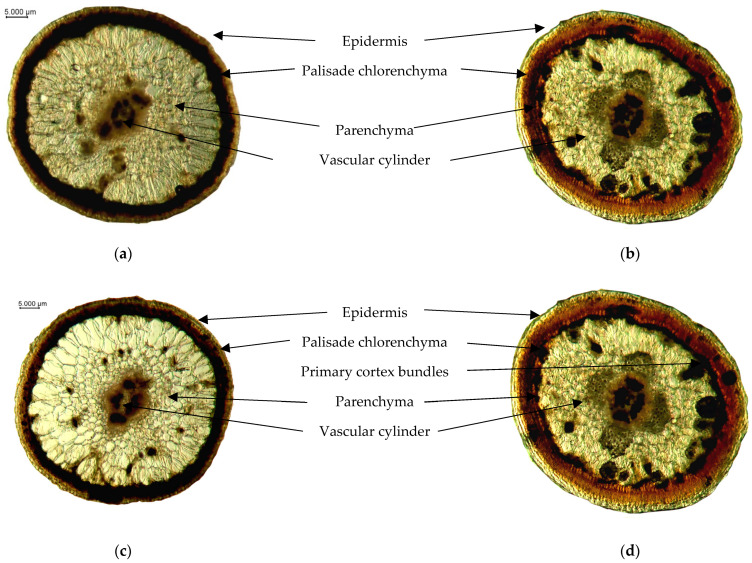
Anatomical structures of control (**a**,**b**) and damaged (**c**,**d**) middle-aged saxaul shoots—(**a**,**c**) *H. aphyllum* and (**b**,**d**) *H. persicum*.

The anatomical structure of the shoots of middle-aged (15–20 years) plants of *H. aphyllum* and *H. persicum* is presented in Figure 1.

**Figure 2:**
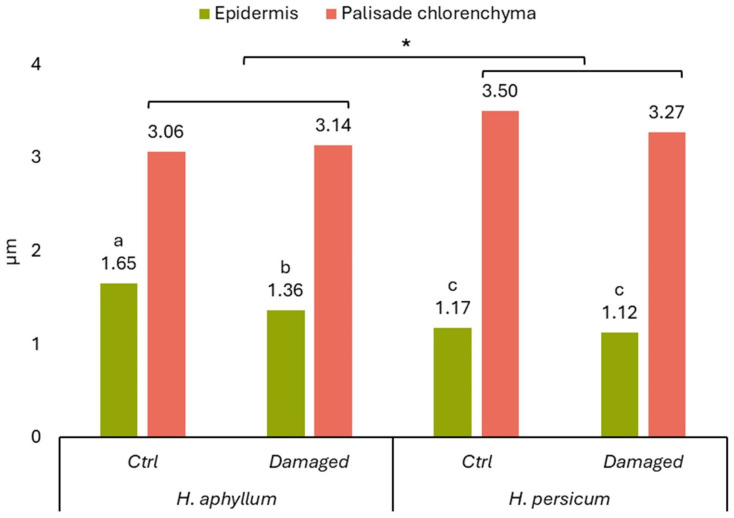
Comparative indicators of epidermal (*p* < 0.05) and palisade chlorenchyma (*p* < 0.05) thickness in middle-aged shoots of *H. aphyllum* and *H. persicum* depending on damage by gall-forming insects. Different letters indicate a significant difference. Notes: * shows significant difference between *Haloxylon* species.

The exterior of saxaul shoots was enveloped by a cuticle. The epidermis of *H. aphyllum* comprised a single layer, while *H. persicum* exhibited 2–3 layers. The data in Figure 2 indicated that the epidermis thickness of middle-aged, undamaged *H. persicum* shoots was significantly thinner compared to that of *H. aphyllum*. Damage from gall-forming insects did not substantially alter the thickness in *H. persicum*, in stark contrast to the notable changes observed in *H. aphyllum*.

Beneath the epidermal cells lies a layer of palisade chlorenchyma. According to Figure 2, the thickness of the palisade chlorenchyma in middle-aged plants of both species remains largely unchanged when affected by gall-forming insects. However, the palisade chlorenchyma in undamaged *H. persicum* shoots was thicker than that in *H. aphyllum* (Figure 2).

**Figure 3:**
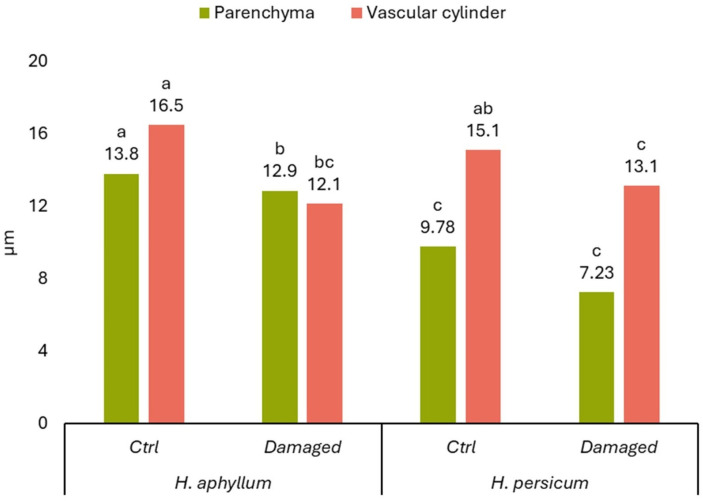
Comparative indicators of parenchyma thickness (*p* < 0.05) and the diameter of the vascular cylinder (*p* < 0.05) in middle-aged shoots of *H. aphyllum* and *H. persicum* depending on damage by gall-forming insects. Different letters within one parameter show significant difference.

Regarding the parenchyma tissue, middle-aged *H. persicum* shoots had significantly thinner tissue compared to *H. aphyllum*. Damage by gall-forming insects led to a marked decrease in the thickness of the parenchyma tissue in *H. aphyllum*, whereas in *H. persicum*, the thickness of the parenchyma tissue remained essentially unchanged when affected by these insects (Figure 3).

Conductive bundles in the central cylinder merge to form a continuous structure. This vascular cylinder comprises both xylem and phloem tissues. In *H. aphyllum*, xylem vessels lacked symmetry and were enclosed by a continuous cambial layer. Conversely, *H. persicum* displayed a symmetrical arrangement of xylem vessels. Figure 3 illustrates the changes in the diameter of the vascular cylinder in middle-aged plants of both *H. aphyllum* and *H. persicum*. Although the diameters of the vascular cylinders in control plants did not significantly differ, the impact of gall-forming insects was more substantial in *H. aphyllum*, showing a notable reduction in the vascular cylinder’s diameter (Figure 3).

These findings highlight that anatomical changes in *Haloxylon* shoots vary with insect damage. Gall-forming insects notably reduced the size of almost all anatomical structures in *Haloxylon* shoots, impacting their water regulation, photosynthesis, and metabolic functions. Damage from gall-forming insects was significantly milder in *H. persicum* compared to *H. aphyllum*, indicating a difference in vulnerability between the two species.

### 2.2. Metabolomic Structure of Intact and Damaged H. aphyllum and H. persicum

In middle-aged *H. aphyllum* shoots, under control conditions, the metabolome composition primarily consisted of fatty acid esters (28.7%), amino acids and their derivatives (21.2%), phytosterols (16.9%), γ-tocopherol (vitamin E) (12.7%), fatty acids (9.73%), carbohydrates and their derivatives (8.25%), phenol derivatives (3.31%), and dialkyl ethers (0.44%), as detailed in Table 1 and Table S1.

For *H. persicum*, the metabolome of middle-aged shoots included fatty acid esters (31.8%), fatty acids (11.9%), carbohydrates and their derivatives (12.4%), terpenoids (10.3%), amino acids and their derivatives (7.21%), phenol derivatives (4.18%), phytosterols (4.72%), γ-tocopherol (vitamin E) (4.99%), and aromatic acid derivatives (3.57%).

**Figure 4:**
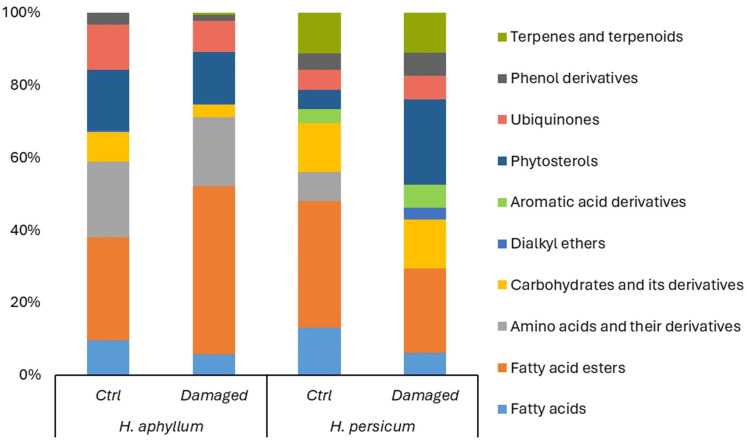
Metabolic spectra of biologically active compounds in middle-aged shoots of *H. aphyllum* and *H. persicum*.

When *H. aphyllum* was damaged by gall-forming insects (Figure 4), there was an observed increase in the content of carbohydrates (notably sucrose) and terpenes (with phytol detected, which was absent in the undamaged variant). Additionally, there was an elevation in the levels of ubiquinones (vitamin E), phytosterols (γ-Sitosterol), phenols (2-Methoxy-4-vinylphenol), and amino acid derivatives (N,N-Dimethylglycine (DMG)). Interestingly, despite these increases, a rise in the level of fatty acids and their esters was also noted, indicating a complex metabolic response to insect damage (Figure 4).

Our findings indicate that in middle-aged (20 years) *H. persicum* plants damaged by gall-forming insects, amino acid derivatives like N,N-Dimethylglycine (DMG) were absent (Figure 4). However, an increase in the content of phenols (specifically 2-Methoxy-4-vinylphenol), phytosterols (γ-Sitosterol), and dialkyl ethers was observed. Notably, in the case of damage, a wider variety of substances was identified, including ethanol, 2-(9-octadecenyloxy)-, (Z)-; benzeneacetonitrile, 4-methoxy-; and benzeneacetonitrile, 4-hydroxy-, whereas only benzeneacetonitrile, 4-hydroxy- was detected in undamaged plants. Concurrently, a decrease in the content of fatty acids and their esters was observed in the damaged plants’ spectrum.

## 3. Discussion

### 3.1. Alterations in the Anatomical Structure of Intact and Damaged H. aphyllum and H. persicum

The interplay between anatomo-morphological traits and developmental patterns is crucial for plant survival and adaptation to environmental constraints, influencing both their geographical distribution and competitive dynamics among related species [37]. Previous morphological analyses indicate that members of the genus *Haloxylon* share many structural similarities in terms of their shoots, leaves, and fruits [38]. It is suggested that *H. persicum* is a genetic derivative of *H. aphyllum* [11]. Morphologically, the primary distinction between these species arises from their preferred habitats. *H. aphyllum* is typically found in ancient river valleys, on stabilized sands, and on takyr-like soils, which might offer somewhat more stable environmental conditions compared to the habitats of *H. persicum*. On the other hand, *H. persicum* thrives in the more challenging conditions of sandy deserts, exhibiting remarkable resilience to extreme temperatures and varying availability of water and light, earning it the designation of a “super-xerophyte” [38,39,40]. The findings presented in Table 2 confirm that *H. aphyllum* and *H. persicum* exhibit similar main morphological parameters when grown under comparable environmental conditions. Despite these morphological similarities, our study also revealed distinct anatomical differences in the shoot structures of these species, which persist across both control plants and those affected by gall-forming insects.

Given the absence of prior comparative anatomical studies on the middle-aged plants of the studied species, it is plausible to suggest that the additional epidermal layers in *H. persicum* not only enhance the plant resistance to water loss but also augment shoot stiffness, thereby acting as a deterrent against potential pests. The superior thickness of the palisade chlorenchyma in *H. persicum* reduced leaf blades, compared to *H. aphyllum*, which further highlights its enhanced adaptability to adverse environmental conditions. The well-developed palisade tissue equips plants to endure stress. Regarding the parenchyma tissue of the studied species, the parenchyma of middle-aged *H. persicum* exhibits a heightened susceptibility to damage from gall-forming insects, in contrast to *H. aphyllum*, which shows robust adaptability in this respect. This is significant because the parenchyma tissue serves as a principal reservoir for water and nutrients [41], crucial for the plant’s sustenance, especially given its limited photosynthetic surface area. However, due to the universal nature of the primary morphophysiological responses to stress, *H. persicum* can be considered an exemplary model for studying plant adaptation mechanisms to diverse stressors [41,42,43].

### 3.2. Alterations in the Metabolomic Structure of Intact and Damaged H. aphyllum and H. persicum

Plant resilience to stress encompasses a complex array of biological responses, including the nuanced regulation of the metabolome, characterized by the synthesis of a broad spectrum of biologically active compounds [44]. These include primary metabolites like carbohydrates, lipids, amino acids, nucleic acids, and organic acids, as well as secondary metabolites such as alkaloids, phenols, quinones, and terpenes [45,46]. These substances are pivotal in shielding plants against adverse abiotic and biotic environmental factors and in ensuring cellular stability [47,48]. Our understanding of the extensive range of sensory signals that govern plant interactions with external factors is still evolving. Delving into the mechanisms through which plants perceive, decode, and react to these signals remains a burgeoning field of research [49].

#### 3.2.1. Fatty Acids and Fatty Acid Esters

Research suggests that one adaptation mechanism of *H. persicum* to arid environments involves an increase in organic acids and their derivatives, particularly various esters [50]. This is significant because fatty acids and their derivatives play a crucial role in modulating membrane properties under stress, particularly affecting membrane fluidity [51,52]. In the control samples of *H. persicum*, there was a notably high concentration of fatty acids and their derivatives. However, in plants damaged by gall-forming insects, a decrease in fatty acid content was observed in both studied species, with an overall increase in the concentrations of their derivatives noted only in *H. aphyllum*.

The literature indicates that stress-resistant plants may ramp up fatty acid production, which beneficially impacts reactive oxygen species (ROS) and modulates abiotic stress signaling [29,53,54]. Yet, the impact of biotic stress, such as that induced by gall formers, on whether the accumulation of fatty acids is beneficial or detrimental remains unclear. Fatty acid metabolism can also signal stress responses, where oxidative stress can transform polyunsaturated fatty acids into various active, often plant-toxic, electrophilic species [55,56]. Interestingly, hexadecanoic acid esters, which occurred at higher concentrations in damaged *H. persicum* plants and lower concentrations in *H. aphyllum*, exhibit larvicidal properties [57,58,59]. Similarly, the ethyl ester of linoleic acid, identified in damaged *H. persicum* plants, exhibits toxicity toward insects [60]. Further, an increase in the levels of 9,12,15-octadecatrienoic acid and its derivatives, as well as 9,12-octadecadienoic acid esters (Z,Z)-, cited as potent antioxidants [61,62,63], in damaged *H. persicum* plants was observed in the current study. Octadecadienoic acid derivatives have also been implicated in the inhibition of snake venom PLA2 toxins and are noted for their significant antimicrobial effects [64,65].

#### 3.2.2. Dialkyl Ethers

In the current study, two compounds from this category were identified. Ethanol, 2-(9-octadecenyloxy)-, (Z)- was found in both control and damaged *H. aphyllum*, with its concentration remaining relatively stable under biotic stress conditions. Conversely, ethanol, 2-(9,12-octadecadienyloxy)-, (Z,Z)- was exclusively detected in *H. persicum* damaged by gall-inducing insects. These compounds, polyunsaturated hydrocarbons often found in aromatic plants, are known for their anti-inflammatory and antioxidant properties [66]. The observed increase in antioxidant activity, attributed to this substance, suggests a heightened ability of *H. persicum* to counter biotic stresses induced by gall-forming insects.

#### 3.2.3. Amino Acids and Their Derivatives

Amino acids and their derivatives are vital for maintaining intracellular osmoregulation and preserving protein structure integrity [67]. The N,N-Dimethylglycine (DMG) identified in our experiments can emerge both during the synthesis of glycine betaine from glycine and its catabolism [68]. According to the literature, DMG may enhance resistance to temperature stress—both high and low—but does not serve as an effective defense against osmotic stress [69,70]. Typically, an increase in amino acid content within plant tissues under stress might be attributed to either a reduction in protein synthesis or an uptick in protein degradation [71].

However, our findings revealed a decrease in DMG concentration in *H. aphyllum* damaged by gall formers, and it was absent in the spectrum of damaged *H. persicum*. This observation underscores the differential capacity of the two saxaul species to withstand biotic stresses. Yet, it remains challenging to assert the significance of DMG in this context.

#### 3.2.4. Carbohydrates and Their Derivatives

Sucrose, synthesized exclusively by photosynthetic organisms, is the product of photosynthesis and serves as the principal transport form of assimilates. It facilitates the movement of reduced carbon and energy throughout the plant. Typically, the accumulation of carbohydrates in leaves is attributed to a decrease in their export, often due to a reduction in photosynthesis [72,73]. However, the increase in sucrose concentration observed in the current study in damaged *H. persicum* may also relate to its role in stabilizing membrane structures and lipid and protein molecules. Additionally, sucrose can neutralize ROS and participate in signaling and metabolic processes, serving as a source of energy and precursors for the synthesis of other protective compounds [74].

Levoglucosan, an organic compound characterized by a six-carbon ring structure, is generated through the pyrolysis of carbohydrates like starch and cellulose, posited to be a key component of plant cell walls [75]. Our observations reveal its absence in *H. aphyllum*, whereas *H. persicum* exhibits a notably high content of levoglucosan in both control and damaged plants, albeit with a slight reduction under the conditions of gall formation. This suggests that levoglucosan, by reinforcing cell walls, could play a crucial role in *H. persicum* defense against damage caused by gall-forming insects.

#### 3.2.5. Aromatic Acid Derivatives

Benzeneacetonitrile derivatives, identified in both control and damaged *H. persicum*, showed increased concentrations following plant damage while being notably absent in *H. aphyllum*. Phenylacetonitrile, a volatile compound released by plants, acts similarly to a phenylalanine-derived pheromone [76,77] and has been observed in microbial processes as well [78]. This de novo synthesis, triggered by insect feeding or external factors like airborne methyl jasmonate [76], indicates an active defense mechanism. Benzeneacetonitrile, a secondary metabolite classified within the benzyl cyanides group, primarily deters insects through the generation of toxic cyanide or hydrogen cyanide ions (https://hmdb.ca/metabolites/HMDB0034171, accessed on 9 April 2024).

Helms et al. [79,80] demonstrated that plants responding to the sex pheromone of the gall-inducing fly *Eurosta solidaginis* exhibited a pronounced induction of jasmonic acid, a protective phytohormone. This response was accompanied by the increased production of damage-associated volatiles and resulted in reduced plant damage in both laboratory and field studies. Consequently, the production of benzeneacetonitrile derivatives plays a crucial role in the *H. persicum* defense strategy.

#### 3.2.6. Phytosterols

Phytosterols, crucial structural components of plant cell membranes, play significant roles in modulating their fluidity and permeability. Cycloartenol, the primary precursor for sterol biosynthesis, is synthesized from squalene by the enzyme cycloartenol synthase (CAS) [81,82]. Therefore, the observed increase in phytosterol levels under stress conditions in studied saxaul species suggests their involvement in fortifying plant cell membranes. Moreover, the variety of phytosterol forms and their concentrations were notably higher in damaged *H. persicum*.

#### 3.2.7. γ-Tocopherol (Vitamin E)

Tocopherols, acting as antioxidants, play a crucial role similar to polyphenols in halting the initial stage of oxidation in cell membranes. Due to their labile hydroxyl group, tocopherols can readily react with hydrocarbon peroxide radicals [83]. Their concentrations are known to increase under moderate stress, a situation where plant damage does not result in fatality [84]. In the current study, tocopherol levels decreased in damaged *H. aphyllum*, while *H. persicum* showed an increase in tocopherol concentration under similar conditions. Notably, the overall tocopherol content was significantly higher in both the control and damaged *H. aphyllum*.

#### 3.2.8. Phenols

Galls are essentially abnormal plant growths triggered by a specific response to the invasion and activities of foreign organisms [85,86]. Research on galls across various plant species has revealed that the chemical composition and concentration of substances within these structures, as well as in the affected plant tissues, can significantly deviate from those in unaffected parts [87,88,89]. Tissues surrounding the gall accumulate high levels of phenolics, tannins, and other compounds, which might be related to protecting the gall as well as the gall-inducing insect [89,90,91,92]. However, this notion is not universally agreed upon in scientific literature [93].

Despite the specifics of the interaction between gall-inducing insects and their plant hosts, evolutionary pressures have shaped both to develop defensive mechanisms against each other [94]. Phenolic compounds play a crucial role in plants, particularly in redox processes, offering antioxidant and anti-stress effects that stabilize cell membranes, prevent mitochondrial autolysis, quench free radicals, and inhibit lipid peroxidation, thereby providing cytoprotective effects [95]. These protective reactions tend to be more pronounced in stress-resistant plant varieties. Mechanical damage to plant tissues often triggers the intensive synthesis of phenolic compounds, leading to oxidative condensation in the outer layers and the formation of a protective barrier, which is more extensive in resistant plants. In our study, a notable decrease in the concentration of 2-Methoxy-4-vinylphenol in *H. aphyllum* damaged by gall inducers contrasted sharply with a significant increase in the same compound in damaged *H. persicum*, where phenol levels were three times higher.

#### 3.2.9. Terpenoids

Terpenes, among numerous biologically active compounds, have demonstrated significant potential in insect control [96,97,98,99], positioning them as promising candidates for natural insecticides [100]. They possess dual functions, acting both as attractants and exerting direct toxic effects on insects [101]. Furthermore, the amphipathic nature of terpenes facilitates hydrophobic interactions between membrane proteins and lipids [102], thus safeguarding against membrane and protein degradation. Terpenoids also play a crucial role in scavenging singlet oxygen generated during oxidative stress [103].

In our study, the overall terpene content in both control and damaged plants remained relatively constant. However, a notable difference lies in the qualitative composition of terpenes between the species. Squalene, phytol, and phytol acetate were identified in *H. persicum*, underscoring a more diverse terpene profile compared to *H. aphyllum*. The literature supports that both phytol and its derivatives [104,105,106], as well as squalene [107,108,109], exhibit marked insecticidal properties. These terpenes are present in the metabolic spectrum of the control *H. persicum* but absent in *H. aphyllum*. Upon damage, squalene was detectable in *H. aphyllum*, albeit in significantly lower quantities than in *H. persicum*, and phytol and its derivatives were completely absent. This disparity in terpene content and diversity could be a key factor underlying the greater vulnerability of *H. aphyllum* to gall-forming insects.

Thus, the current research concludes that the metabolic pathways contributing to resistance against gall-inducing insects in *H. aphyllum* are primarily focused on the biosynthesis of fatty acids and their derivatives and the biosynthesis of γ-tocopherol (vitamin E). In contrast, *H. persicum* engages a broader spectrum of biosynthetic pathways, including those for fatty acids and their derivatives, dialkyl ethers, carbohydrates and their derivatives, aromatic acid derivatives, phytosterols, γ-tocopherol (vitamin E), phenols, and terpenoids.

According to the literature, the most successful plant species in resisting pests often synthesize a wide array of moderately toxic protective compounds or a small amount of highly toxic substances [110]. The effectiveness and role of different metabolites in response to biotic stresses may vary between saxaul species, likely due to the distinct gene regulatory networks in each species. A diversity of biologically active secondary metabolites tends to offer more effective protection against insects than a single potent compound, and the varying physical properties of these substances can ensure more durable defense mechanisms [110,111,112].

Hence, our findings indicate that the modulation of metabolic pathways plays a significant role in enhancing the survival and growth of *H. persicum* under biotic stress conditions. There is a compelling case for further in-depth studies on the metabolic profile of *H. persicum* to isolate and purify bioactive substances, given their potent antioxidant and insecticidal properties. The array of phytochemicals identified in *H. persicum* has the potential to be utilized in developing new insecticides targeting specific or multiple aspects of insect physiology, thereby promoting economic and environmental sustainability [110,113].

## 4. Materials and Methods

### 4.1. Research Objects and Plant Collection Sites

The saxaul species under study, characterized as shrubs or small trees, exhibit forked branching and jointed, brittle young shoots. *H. aphyllum* was distinguished by leaves reduced to tubercles, serving its photosynthetic needs alongside assimilating branches [114] that contain chlorophyll.

*H. aphyllum* was found in the pre-sand zone, characterized by desert sandy crust soil, and served as the community edifier within a plant community comprising forbs, white earth wormwood, and black saxaul on sandy terrains. These trees, aged between 15 and 20 years, had an average height of 1.5 ± 0.08 m and a trunk diameter of 7 ± 0.9 cm, with a crown spanning approximately 1.48 ± 0.12 × 1.39 ± 0.12 m. The spacing between individual *H. aphyllum* plants averaged 2.84 ± 0.21 m, and the total projective cover of the plant community was 40%. Notably, over 50% of *H. aphyllum* trees at the collection site were infested by gall-forming pests, predominantly by species such as *Caillardia azurea* [25], *Asiodiplosis noxia* [115], *Caillardia robusta* [25], and *Aceria haloxylonis* [21].

Conversely, *H. persicum* thrived in dune zones and hummocky ridges, where the soil also featured a desert sandy crust. As the community edifier, it forms part of a forb–white earth wormwood–white saxaul plant community on sandy desert soils. These plants, aged between 15 and 20 years, stand at an average height of 1.3 ± 0.1 m, with a trunk diameter of 3.1 ± 0.6 cm, and their crowns measure roughly 1.16 ± 0.14 × 1.09 ± 0.22 m. *H. persicum* plants were typically spaced 2.03 ± 0.2 m meters apart, and the total projective plant cover in this habitat reached 50%. The degree of infestation by gall-forming pests in *H. persicum* was comparatively lower, up to 25%, with dominant insect species including *Asiodiplosis ulkunkalkani* [115], and *Caillardia notata* [25]. The characteristics of collection sites and plant parameters are summarized in Table 2.

### 4.2. Anatomical Structure Analysis

The conservation of plant material was conducted using the Strasburger–Flemming method [116]. The process began with fixation in 70% ethanol, ensuring the preservation of the fixed material by storing it in a mixture composed of equal parts ethanol, glycerol, and water (1:1:1 ratio).

For anatomical examination, preparations were made using a microtome fitted with a TOS-2 freezing unit. These sections, with thicknesses ranging from 10 to 15 microns, were then treated with glycerin and balsam, adhering to the traditional methodologies established by Barykina [116]. The anatomical sections were analyzed by microphotography, utilizing a microscope equipped with an MC 300 CAMV400/1.3M camera (Micros, Vienna, Austria).

### 4.3. Metabolomic Analysis

The analysis of organic biologically active compounds in *Haloxylon* spp. was conducted using gas chromatography coupled with mass spectrometric (GC-MS) detection (Agilent 6890N/5973N, Santa Clara, CA, USA). Plant tissue samples were initially preserved in 96% ethanol, using a ratio of 100 g of tissue to 500 mL of ethanol. The extraction process involved two stages of 72 h each on an orbital shaker, using the same solvent, until the solvent appeared clear and colorless. For the analysis, 1.0 µL of each sample was injected into the GC-MS system at an injection temperature of 260 °C, with no split flow. To ensure reliability, each sample underwent a single injection, with a total of three technical replicates processed.

The separation of compounds was achieved using a DB-35MS (Agilent Technologies, Santa Clara, CA, USA) capillary chromatographic column (30 m in length, an internal diameter of 0.25 mm, and a film thickness of 0.25 µm), with helium as the carrier gas at a constant flow rate of 1 mL min^−1^. The temperature program started at 40 °C (no hold time) and increased to 150 °C at a rate of 10 °C min^−1^ (no hold time), followed by a ramp up to 300 °C at 5 °C min^−1^ (with a 10-min holding time). Detection was performed in SCAN *m*/*z* 34-850.

The GC system was managed using Agilent MSD ChemStation software (version 1701EA, Agilent Technologies, Santa Clara, CA, USA) for controlling the system, recording, and processing the results. Data analysis included the determination of retention times, peak areas, and analysis of spectral information from the MS detector. The interpretation of the mass spectra utilized the Wiley 7th edition and NIST’02 libraries, which contain over 550,000 spectra.

The data analysis was conducted using Rstudio software (version 2023.06.0 Build 421, RStudio PBC, 2023). Tukey HSD tests were performed for the pairwise comparisons of the means, while ANOVA was used to confirm statistical significance. Subsequently, the treatments were categorized by a letter in descending order, and graphs were generated. The significance was declared at *p* < 0.05.

## 5. Conclusions

The study findings revealed that gall-forming insects significantly impact the anatomical structures of *Haloxylon* shoots, with *H. persicum* experiencing less severe damage compared to *H. aphyllum*. It was observed that the key metabolic pathways contributing to resistance against gall inducers in *H. aphyllum* were centered around the biosynthesis of fatty acids and their derivatives and the biosynthesis of γ-tocopherol (vitamin E). In contrast, *H. persicum* engaged a broader array of biosynthetic pathways, including fatty acids and their derivatives, dialkyl ethers, carbohydrates and their derivatives, aromatic acid derivatives, phytosterols, γ-tocopherol (vitamin E), phenols, and terpenoids.

Indeed, the findings obtained highlight the importance of metabolic pathway modulation in enabling *H. persicum* to withstand biotic stresses more effectively, specifically safe plant survival and growth. The diverse metabolic profile of *H. persicum* requires further in-depth investigation for the extraction and isolation of biologically active compounds, with potent antioxidant and insecticidal properties, holding significant promise for pharmacological and industrial applications. Moreover, the array of phytochemicals identified in *H. persicum* has the potential for development into innovative insecticides that target specific or multiple pests, contributing to economic and environmental sustainability.

## Figures and Tables

**Table 1:** Biologically active compounds (%) content in the metabolic spectra of undamaged and damaged plants of *H. aphyllum* and *H. persicum*.

Metabolite Classes	*H. aphyllum*		*H. persicum*	
	Ctrl	Damaged	Ctrl	Damaged
Fatty acids				
Hexadecanoic acid	9.73 ± 0.68	5.72 ± 0.74 *	5.46 ± 0.42	-
9,12-Octadecadienoic acid (Z,Z)-	-	-	1.94 ± 0.53	-
9,12,15-Octadecatrienoic acid, (Z,Z,Z)-	-	-	4.54 ± 0.23	5.44 ± 0.71
Fatty acid esters				
Hexadecanoic acid, methyl	4.89 ± 0.47	1.92 ± 0.28 *	2.81 ± 0.29	4.39 ± 0.87 *
Hexadecanoic acid, octadecyl	1.65 ± 0.17	-	-	-
8-Octadecenoic acid, methyl	-	0.73 ± 0.13	-	-
9-Octadecenoic acid, methyl (E)-	1.67 ± 0.14	-	2.09 ± 0.31	2.84 ± 0.80
12-Octadecadienoic acid (Z,Z)-, methyl	-	1.61 ± 0.25 *	-	-
9,12-Octadecadienoic acid (Z,Z)-, methyl	3.36 ± 0.19	-	3.21 ± 0.24	4.56 ± 0.55 *
9,12,15-Octadecatrienoic acid, methyl, (Z,Z,Z)-	5.79 ± 0.18	2.62 ± 0.34 *	-	5.53 ± 0.76 *
9,12,15-Octadecatrienoic acid, ethyl, (Z,Z,Z)-	2.43 ± 0.04	1.03 ± 0.03 *	-	1.55 ± 0.10 *
9,12,15-Octadecatrienoic acid, 2,3-dihydroxypropyl, (Z,Z,Z)-	8.92 ± 0.16	4.70 ± 0.57 *	-	-
Olean-12-en-28-oic acid, 3-oxo-, methyl	-	-	17.0 ± 0.89	-
Olean-12-en-28-oic acid, 2,3,23-trihydroxy-, methyl, _(2α,3β,4α)-_	-	10.1 ± 1.52 *	-	-
Olean-12-en-28-oic acid, 3-(acetyloxy)-, methyl, (3β)-	-	25.1 ± 1.55 *	6.63 ± 0.59	-
Linoleic acid ethyl	-	-	-	1.56 ± 0.40 *
Dialkyl ethers				
Ethanol, 2-(9-octadecenyloxy)-, (Z)-	0.44 ± 0.13	0.40 ± 0.09	-	-
Ethanol, 2-(9,12-octadecadienyloxy)-, (Z,Z)-	-	-	-	3.02 ± 0.29 *
Amino acids and their derivatives				
N,N-Dimethylglycine	21.2 ± 3.60	18.8 ± 3.64	7.21 ± 0.15	-
Carbohydrates and derivatives				
Sucrose	8.25 ± 0.35	3.41 ± 0.34*	1.92 ± 0.18	2.62 ± 0.12 *
β-D-Glucopyranose, 1,6-anhydro- (levoglucosan)	-	-	10.5 ± 0.85	9.26 ± 0.79
Aromatic acid derivatives				
Benzeneacetonitrile, 4-methoxy-	-	-	-	1.47 ± 0.11 *
Benzeneacetonitrile, 4-hydroxy-	-	-	3.57 ± 0.28	3.99 ± 0.25
Phytosterols				
γ-Sitosterol	16.9 ± 1.82	14.1 ± 0.17*	4.72 ± 0.23	7.19 ± 0.12 *
β-Sitosterol acetate	-	-	-	4.70 ± 0.58 *
Stigmasterol	-	-	-	8.81 ± 0.98 *
γ-tocopherol (vitamin E)				
γ-tocopherol (vitamin E)	12.7 ± 0.95	8.55 ± 0.64 *	4.99 ± 0.43	5.80 ± 0.32
Phenol derivatives				
2-Methoxy-4-vinylphenol	3.31 ± 0.37	1.67 ± 0.07 *	4.18 ± 0.16	5.56 ± 0.36 *
Terpenoids				
Squalene	-	0.89 ± 0.09 *	2.63 ± 0.13	3.21 ± 0.20 *
Phytol	-	-	6.85 ± 0.54	6.68 ± 0.46
Phytol, acetate	-	-	0.92 ± 0.06	- *

Notes: * represents significant differences at *p* ≤ 0.05 and *n* = 10 plants in each of the 3 replicates for all treatments.

**Table 2:** Collection sites and taxation plant profile.

Collection Site		Taxation Profile			Damage
GPS	Location	Height, m	Stem Diameter, cm	Crown Diameter, m	
*H. aphyllum*					
N 44°29′40″ E 62°22′59″	Kyzylorda region, Kazalinsky district, 57 km southeast of the village Azhar	1.2	2.9	1.15	yes
		1.3	3.2	1.65	no
		1.5	4.0	1.85	no
		1.5	4.2	1.80	no
		1.6	3.8	1.45	yes
		1.7	5.5	1.75	yes
*H. persicum*					
N 44°55′32″ E 62°15′15″	Kyzylorda region, Kazalinsky district, 89 km south of Kazalinsk	1.3	3.5	1.35	yes
		1.3	5.7	1.80	yes
		1.3	7.1	1.85	no
		1.4	3.4	1.45	no
		1.4	3.8	1.10	yes
		1.6	4.4	1.25	no

## Data Availability

The data presented in this study are available on request from the corresponding author.

## Supplementary Materials

The following supporting information can be downloaded at: https://www.mdpi.com/article/10.3390/ijms25094738/s1.
